# Functional analysis of *TWIST1* domains regulating smooth muscle cell phenotype

**DOI:** 10.3389/fcvm.2025.1659847

**Published:** 2025-10-31

**Authors:** Danielle C. M. Dy, Thiel Lehman, Adrian Othon, Mitesh Rathod, William J. Polacheck, Robert Wirka

**Affiliations:** ^1^ Department of Cell Biology & Physiology, University of North Carolina at Chapel Hill, Chapel Hill, NC, United States; ^2^ McAllister Heart Institute, University of North Carolina at Chapel Hill, Chapel Hill, NC, United States; ^3^ Department of Pharmacology, University of North Carolina at Chapel Hill, Chapel Hill, NC, United States; ^4^ Lampe Joint Department of Biomedical Engineering, University of North Carolina at Chapel Hill and North Carolina State University, Chapel Hill and Raleigh, NC, United States; ^5^ Department of Medicine Division of Cardiology, University of North Carolina at Chapel Hill, Chapel Hill, NC, United States

**Keywords:** TWIST1, GWAS, atherosclerosis, hypertension, smooth muscle cells, phenotypic modulation, cardiovascular

## Abstract

**Introduction:**

TWIST1, a bHLH transcription factor, regulates mesenchymal specification, differentiation, proliferation and migration during development and in diseases such as cancer. More recently, genome-wide association studies have identified TWIST1 as a causal gene that increases risk for multiple vascular diseases, including atherosclerosis and hypertension. However, its molecular role in the vascular wall remains unclear.

**Methods:**

In this study, we interrogated how TWIST1 dimer composition and discrete TWIST1 domains affect SMC phenotype by expressing forced TWIST1 dimers or TWIST1 variants lacking specific domains, followed by bulk RNA sequencing and proliferation and migration assays in human coronary artery SMCs (HCASMCs).

**Results:**

We found that TWIST1 homodimers had only modest transcriptomic effects but strongly promoted migration and proliferation–effects abolished by deletion of the TWIST1 N-terminus. Heterodimerization of TWIST1 with TCF3-encoded E proteins resulted in larger transcriptomic effects, promoting Rho/ROCK signaling and extracellular matrix production/organization, but had only modest effects on proliferation and no effect on migration. Deletion of the TWIST1 C-terminus resulted in a very large transcriptomic shift with predicted downregulation of angiotensin and Rho/ROCK signaling as well as ECM production/organization pathways, in a manner suggesting a dominant negative effect on TWIST1-E12 function. Comparison with single-cell RNA-seq data from human endarterectomy samples placed the function of these TWIST1 variants in a disease context and showed that deletion of the C-terminal domain prevented a modulated SMC phenotype.

**Discussion:**

These studies demonstrate that TWIST1 influences different aspects of SMC phenotype independently via discrete domains and dimer composition, and link TWIST1 to key signaling pathways that influence SMC phenotype during disease.

## 1 Introduction

TWIST1 is a basic helix-loop-helix (bHLH) transcription factor that functions as a master regulator of mesoderm specification during development (1–3). It has also been identified as a key regulator of cellular processes including epithelial-to-mesenchymal transition (EMT), which is characterized by the acquisition of a mesenchymal phenotype and a concomitant increase in cellular proliferation and migration (4). TWIST1-mediated EMT has been shown to be important in the context of various cancers (5, 6), and mutations in the *TWIST1* gene are also associated with the genetic developmental disorder Saethre-Chotzen syndrome (SCS) (7). More recently, *TWIST1* has been identified by Genome Wide Association Studies (GWAS) as a causal gene that mediates risk for coronary artery disease (CAD) (8) as well as various vascular disorders including ischemic heart disease, stroke, Moyamoya disease, hypertension and peripheral artery disease (PAD) (8–12). Specifically, the risk allele (rs2107595-T) for all of these vascular diseases is associated with increased *TWIST1* expression in GTEx, indicating that *TWIST1* expression promotes disease risk. To understand TWIST1's role in vascular disease, it is crucial to determine how its gene regulation program alters cell phenotype in relevant vascular wall cell types.

TWIST1 is known to act as a dimer—either as a homodimer or as a heterodimer with the E proteins E12 or E47 (both encoded by the gene TCF3). The activity of TWIST1 has been shown to differ depending on whether it forms a homo- or hetero-dimer (13), and this dimerization mediated by the **α**-helices in its bHLH domain (14, 15). The N -terminus of TWIST1 contains 2 nuclear localization sites (NLS) that interact with p300, cAMP-response element binding protein (CREB) and CREB-binding protein (CBP) (1), resulting in the inhibition of acetyltransferase activities of these histone remodeling enzymes (1, 16). TWIST1's C-terminus contains a domain known as the ‘WR motif’ or the ‘Twist1 box’ and has been shown to interact with members of the MADS box transcription enhancer factor 2 (MEF2) as well as the transcription factor RUNX2 (1, 17–21). Previous studies have suggested that the C-terminus is necessary for proper TWIST1 protein folding and activity (1, 21). Therefore, individual domains of TWIST1 mediate its dimerization and its interaction with known binding partners, but the effect of these domains on TWIST1's function in smooth muscle cell (SMC) phenotype are unknown. In this study, we performed functional analysis of TWIST1 dimer composition and functional domains to elucidate their role in altering SMC phenotype. We found that TWIST1 heterodimerization with E-proteins drives transcriptional shifts that promote extracellular matrix (ECM) remodeling with modest effects on SMC proliferation. In contrast, TWIST1 homodimers induce a strongly migratory and proliferative SMC phenotype with minimal transcriptional reprogramming. Domain-mapping studies reveal that the TWIST1 N-terminus primarily mediates its effects on proliferation and migration, whereas the C-terminus is required to promote ECM production and organization. Finally, we identify a novel link between TWIST1 activity and Rho/ROCK signaling, suggesting a mechanistic basis for its role in vascular disease risk.

## 2 Methods

### 2.1 Cloning of dimer overexpression constructs into pWPI

The pWPI plasmid was obtained from Addgene (12254), as well as sequences for E12 and E47 in plasmids 58492 and 16059, respectively. Human *TWIST1* was obtained from Origene (NM_000474 Human Tagged ORF Clone, SKU RC202920; Origene). The 81-base pair linker sequence was 5’- GGAGGCGGCAGCTCCGGCGGAAGCGGCGGAGGCTCTGGAGGAGGATCTTCTGGCGGATCTGGCGGAGGATCTGGAGAGTTT-3’. A myc-tag sequence (5’- GAACAAAAGTTGATTTCTGAAGAAGATTTG-3’) was also cloned at the start of each overexpression sequence.

The linker sequence was first cloned into the pWPI plasmid at the Pme1 (R0560S; New England Biolabs) site using HiFi DNA Assembly (E2621l; New England Biolabs). This pWPI-linker construct served as the base for each of the dimer constructs.

As all dimer constructs contained the *TWIST1* monomer, a myc-tagged TWIST1 fragment was cloned into the pWPI-linker vector. The TWIST1 fragment was amplified using Q5 High-Fidelity DNA Polymerase (M0491S; New England Biolabs) with the 5’-end including the c-myc sequence, followed by gel extraction. The pWPI-linker backbone was digested using BamH1-HF enzyme (R3136S; New England Biolabs) and the gel-extracted myc-TWIST1 fragment was cloned in via HiFi Assembly.

This myc-TWIST1-linker in pWPI construct was used to clone the 3 different dimer variants using the BamHI enzyme, via HiFi assembly. Primers for the TWIST1 homodimer and heterodimers can be found in Supplementary Table S2. All plasmids were sequence verified.

### 2.2 Cloning of TWIST1 domain variants

Full length TWIST1 and domain variants were amplified from Human *TWIST1* obtained from Origene (NM_000474 Human Tagged ORF Clone, SKU RC202920; Origene) using the primers in Supplementary Table S2. Amplified TWIST1 fragments were cloned using the standard NEB HiFi DNA Assembly protocol (E2621l; New England Biolabs). Fragments were inserted into a NheI-HF (R3131S, NEB) digested pLJM1-EGFP expression vector (19319, Addgene) modified with a T2A linker between the TWIST1 fragment and the EGFP coding region. All TWIST1 and domain variant plasmids were sequence verified.

### 2.3 Cell culture and lentiviral transduction of HCASMCs

Human coronary artery smooth muscle cells (HCASMCs) were ordered from Lonza (CC-2583; Batch 20TL266549) and cultured in Lonza's SmBM Basal medium (CC-3181; Lonza) with SmGM-2 Smooth Muscle Cell Growth Medium-2 Bullet Kit (CC-4149; Lonza), excluding the gentamicin sulfate. HCASMCs were used between passage 5–6 for all experiments.

For bulk RNA-Seq and qPCR experiments, 4 biological replicates for each condition were prepared. HCAMSCs were seeded into 6-well plates and at 70% confluence and serum deprived for 24 h. Following serum deprivation, the media was changed to 1 ml of full-serum SmGM medium, 1 ml of lentivirus, and 2 μl of cell-culture grade polybrene (TR-1003-G; Millipore Sigma) to increase transduction efficiency. After overnight incubation, the medium was replaced with fresh full-serum SmGM medium. After 48 h, HCASMCs were harvested for RNA extraction using the Zymo Quick-RNA MicroPrep Kit (11-327M; Zymo Research).

### 2.4 Bulk RNA-Seq

The DeNovix RNA Fluorescence Assay and Agilent Bioanalyzer RNA Pico Kit (5067-1513; Agilent) were used to evaluate RNA concentration and quality. All samples had a RIN score greater than 9 and a concentration higher than 45 ng/μl. The NEBNext Ultra II Directional Kit (E7760; New England Biolabs) was used for library preparation, and was quantified using the DeNovix DNA Fluorescence Assay and Agilent Bioanalyzer HS DNA Kit. Libraries were sequenced by Admera Health using the NovaSeq X Plus platform.

Average TPM for *TWIST1* was calculated for each condition to validate overexpression of dimer and deletion variants, respectively. The average TPM of *TWIST1* in pWPI-treated HCASMCs was 16.36. TWIST1-E12-treated HCASMCs showed 83-fold increase, TWIST1-E47-treated HCASMCs showed a 69-fold increase, and TWIST1-TWIST1-treated HCASMCs exhibited a 182-fold increase. The same calculations were performed for the dimer variants. PLJM1-treated cells had an average *TWIST1* TPM of 43.59, while TWIST1-treated HCASMCs showed a 592-fold increase, ΔN-treated HCAMSCs showed a 1174-fold increase, ΔbHLH showed a 209-fold increase, and ΔC-treated HCAMSCs exhibited a 2129-fold increase.

### 2.5 Validation of nuclear localization using immunocytochemistry

HCASM cells were seeded onto 12 mm diameter coverslips (501929540, Fisher Scientific) coated with 0.005% Poly-L-Lysine (P4707, Sigma-Aldrich). Cells were allowed to adhere for an hour before half of the media was removed and an equal volume of virus solution and 1 μL of polybrene was added. Cells were incubated overnight at 37°C before the viral solution was removed and fresh media was added. Cells were incubated for an additional 48 h at 37°C before starting the immunocytochemistry protocol.

HCASMC media was removed, and cells were fixed with 4% paraformaldehyde (101176-014, VWR) for 15 min and washed twice with 3% BSA (37520, Fisher Scientific). Next, cells were incubated for 20 min in 0.5% Triton X-100 (A16046AE, Fisher Scientific) before being blocked in 10% goat serum for 30 min (50197Z, ThermoFisher Scientific). After removal of blocking solution and rinse with 3% BSA, cells were incubated with primary anti-body (1:400); Anti-Myc (71D10, Cell Signaling) or Anti-IgG (ab172730, Abcam) in 1% BSA for 2 h. Primary anti-body solution was removed, and cells were washed thrice with 3% BSA for 5 min. Secondary anti-body (1:500) Alexa Fluor 647 (ab150079, Abcam) was added in 1% BSA to cells and incubating for 1 h in the dark. Secondary anti-body solution was removed, and cells were rinsed thrice with 3% BSA for 5 min in the dark. Finally, coverslips were mounted on Frosted Microscope Slides (12-5442, Fisher Scientific) in VECTASHIELD Antifade Mounting Medium with DAPI (NC9524612, Fisher Scientific).

### 2.6 HCASMC functional assays

HCASMCs at 70% confluence were serum deprived for 24 h before lentiviral transduction, as previously described. Following overnight incubation with virus, the media was replaced with full-serum SmGm media and incubated for 48 h to allow for expression of the constructs. After 48 h, the plunger seal of a sterile 1 ml syringe (309628; BD) was dipped into Trypsin 0.025%—EDTA 0.02% in HBSS without Calcium and Magnesium (118-093-721; Quality Biological) and blotted on a sterile plate to remove excess solution. The plunger seal was pressed into the center of a 12-well, creating a circular wound area in the center. This process was repeated for all experimental wells, using a new syringe per well. 1 ml of fresh full-serum SmGm was then added to each well.

To measure the effect of each construct on HCASMC migration, images were taken using an EVOS M7000 Imaging System (AMF7000; ThermoFisher Scientific) every 4 h up to 24 h. To ensure consistency, an auto-scan protocol was used to image the wells at each time point and stitched images were used to analyze the wound area over time.

In addition to quantification of migration of treated HCASMCs, a proliferation assay was performed using the baseclick EdU Cell Proliferation Assay for Imaging in dye 594 (BCK-EdU594IM100; baseclick) at the 12 h post-wound time point. After capturing images at the 24 h post-wound time point for migration quantification, cells were fixed with 4% PFA for 15 min at RT, washed twice with 3% BSA (Fraction V, Culture grade pH 7, Non-sterile; SH30574.02; Cytiva HyClone) in PBS, and permeabilized with 0.5% Triton X-100 (BP151-500; fisher bioreagents) in PBS for 20 min at RT. Following permeabilization, the cells were washed twice with 3% BSA before each well was incubated with 250 μl of the EdU reaction cocktail for 30 min at RT in a dark room. The reaction cocktail was removed, and the cells were washed 3 times with 3% BSA. Finally, cells were incubated with a 1 μg/ml solution of Hoechst 33342 (561906; BD Pharmingen) in PBS for 15 min at RT in a dark room. Following this final incubation, the Hoechst solution was replaced with 1X PBS and cells were imaged for Hoechst, GFP, and EdU signals using the EVOS7000 system. Quantification of colocalized signal was performed on images without the wound and summed to obtain the total proportion of proliferating HCASMCs per well. Quantification analysis was performed using Fiji ImageJ software (ver 1.54f) (22).

Transduction efficiency was quantified by applying a threshold algorithm to each the DAPI and EGFP channels. ImageJ's ‘Image Calculator’ function was used to colocalize signal for both DAPI and EGFP, representing the transduced HCASMCs. Transduction efficiency is represented as the percentage of all DAPI-positive cells that are positive for colocalized signal of DAPI and EGFP. The ‘Analyze Particles’ function in ImageJ was used to quantify nuclei with co-localized signal.

### 2.7 Statistical analysis of functional assays

Statistical analyses of functional assays were performed using GraphPad Prism (version 10.1.1, build 323). For dimer migration, dimer proliferation, and domain mutation variant proliferation functional assays, normality was assessed using the Shapiro–Wilk test for each sample group. For the dimer migration analysis, comparisons between two sample groups within the same time point were conducted using unpaired Welch's *t*-tests. Welch's *t*-tests were also performed for comparisons of the proliferation data. For the domain variant migration assay, the same normality testing was applied. Since multiple groups were compared, statistical significance was evaluated using a one-way ANOVA followed by Tukey's *post hoc* test. All comparisons were restricted to within the same time point.

### 2.8 Processing and analysis of bulk RNA-Seq data

FASTQ files were processed using nf-core/rnaseq (version 3.17.0) pipeline (23). Pre-processing to infer strandedness was performed using fq (v0.12.0) and Salmon (v1.10.3). The reference genome GRCh38.primary_assembly reference genome was used. Reads were trimmed using fastp (v0.23.4) and alignment was performed using STAR (v2.7.10a) and the count matrix data were generated using RSEM (v1.3.1) with a minimum mapped reads value of 5. Count matrix data was processed using DESeq2 (v1.28.0) (24). For Principal Component Analysis of samples, the Transcripts Per Million (TPM) values were normalized using a log2 transformation. Normalized TPM values were also used to perform and visualize Pearson's Correlation and hierarchical clustering analyses.

Differential gene expression analysis was performed using the expected counts of each sample. Prior to DE gene analysis, prefiltering was performed to exclude genes with counts below 10 across fewer than 4 samples, as each condition included 4 replicates. For all analyses, an adjusted *p*-value cut-off of 0.05, and a log2 fold change cut-off of 1.5 was applied. One exception to this application was for the ΔC compared to TWIST1 analysis, where an adjusted *p*-value cut-off of 10^−10^ was applied. The DEG lists were uploaded to QIAGEN's IPA software (25) to determine the top predicted pathways, top predicted upstream regulators, and terms for diseases and functions. To identify candidate genes that may regulate proliferation and migration, the ‘Diseases & Functions’ terms in IPA were used to identify pathways associated with cell movement and proliferation. Common genes within these pathways across multiple data sets were identified, and the log2FoldChange values for these genes in the DEG analysis comparisons from DESeq2 were plotted.

### 2.9 Analysis of human endarterectomy scRNA-Seq data

FASTQ files from Tan, et al (26). were downloaded from biosino.org, accession number OEP00001731, and processed with the BD Rhapsody pipeline (v 2.2.1) to generate cell-by-gene count matrices for each sample. Count matrices were used in Seurat (v5) for all further analyses. Briefly, cells with less than 500 or more than 4,000 features, or more than 15,000 counts were excluded, as were cells with more than 20 percent mitochondrial genes. The top 1000 most highly-variant genes were used for principal component analysis (PCA), followed by shared nearest neighbor (SNN) modularity optimization-based clustering and UMAP visualization. Analysis for differentially-expressed genes between clusters was performed using the ‘FindMarkers’ function using default parameters.

### 2.10 Calculation of SMC phenotypic modulation score

First, genes that were downregulated or upregulated during SMC phenotypic modulation *in vivo* were determined using the FindMarkers function in Seurat, comparing all modulated SMCs (clusters 2, 5, 0) against differentiated SMCs (clusters 4 and 6). This resulted in a ‘*in vivo* SMC modulation list’ in which the log2FC of each gene indicated whether it was up- or down-regulated with SMC modulation. Second, the *in vitro* DEGs for each TWIST1 variant vs. empty vector were intersected with the ‘*in vivo* SMC modulation list’, retaining genes that were differentially regulated both *in vivo* and *in vitro*. Third, because the intent was to create a composite score in which positive values represented movement towards modulated SMCs, the sign of the *in vitro* log2FC values was reversed for all genes that were more highly expressed in differentiated SMCs in the *in vivo* scRNAseq data. For instance, if an *in vitro* variant produced a positive log2FC for *CNN1*, this was multiplied by −1 because, with respect to this gene, it indicated that the TWIST1 variant was promoting a less modulated phenotype, thereby decreasing the summed score. Similarly, a variant producing a negative log2FC for *CNN1* also had this value multiplied by −1, which resulted in a positive value that increased the score, indicating it promoted a more modulated phenotype. These log2FC values were summed for each variant to produce a final composite score (see example in Supplementary Table S1).

### 2.11 qPCR validation

Forty-eight hours after transduction, HCASMCs were harvested for RNA extraction using the Zymo Quick-RNA MicroPrep Kit (11-327M; Zymo Research)**.** An additional gDNA cleanup was performed using the TURBO DNA-free Kit (AM1907; ThermoFisher Scientific). For all samples, and the iScript cDNA Synthesis Kit (1708890; BIO-RAD) was used to generate cDNA.

To quantify the relative changes in gene expression, qPCR reactions were prepared using TaqMan Universal Mastermix II no UNG (4440040; ThermoFisher Scientific) and the following human TaqMan Gene Expression Assays: UBC (4448489; ThermoFisher Scientific), TWIST1 (Hs00361186_m1 4331182; ThermoFisher Scientific), CNN1 (4453320; ThermoFisher Scientific), ACTA2 (HS00426835_g1 4331182; ThermoFisher Scientific), TAGLN (Hs01038777_g1 4453320; ThermoFisher Scientific), DCN (Hs00370385_m1 4448892; ThermoFisher Scientific), TNFRSF11B (Hs00900358_m1 4331182; ThermoFisher Scientific), TMEM119 (Hs01938722_u1 4331182; ThermoFisher Scientific), IBSP (Hs00913377 IBSP 4453320; ThermoFisher Scientific). Three technical qPCR replicates were performed for each sample. The standard protocol for a TaqMan reaction for Comparative CT was run using the Viia6 Flex qPCR machine.

## 3 Results

### 3.1 TWIST1 dimers regulate distinct transcriptional profiles

**Figure 1:**
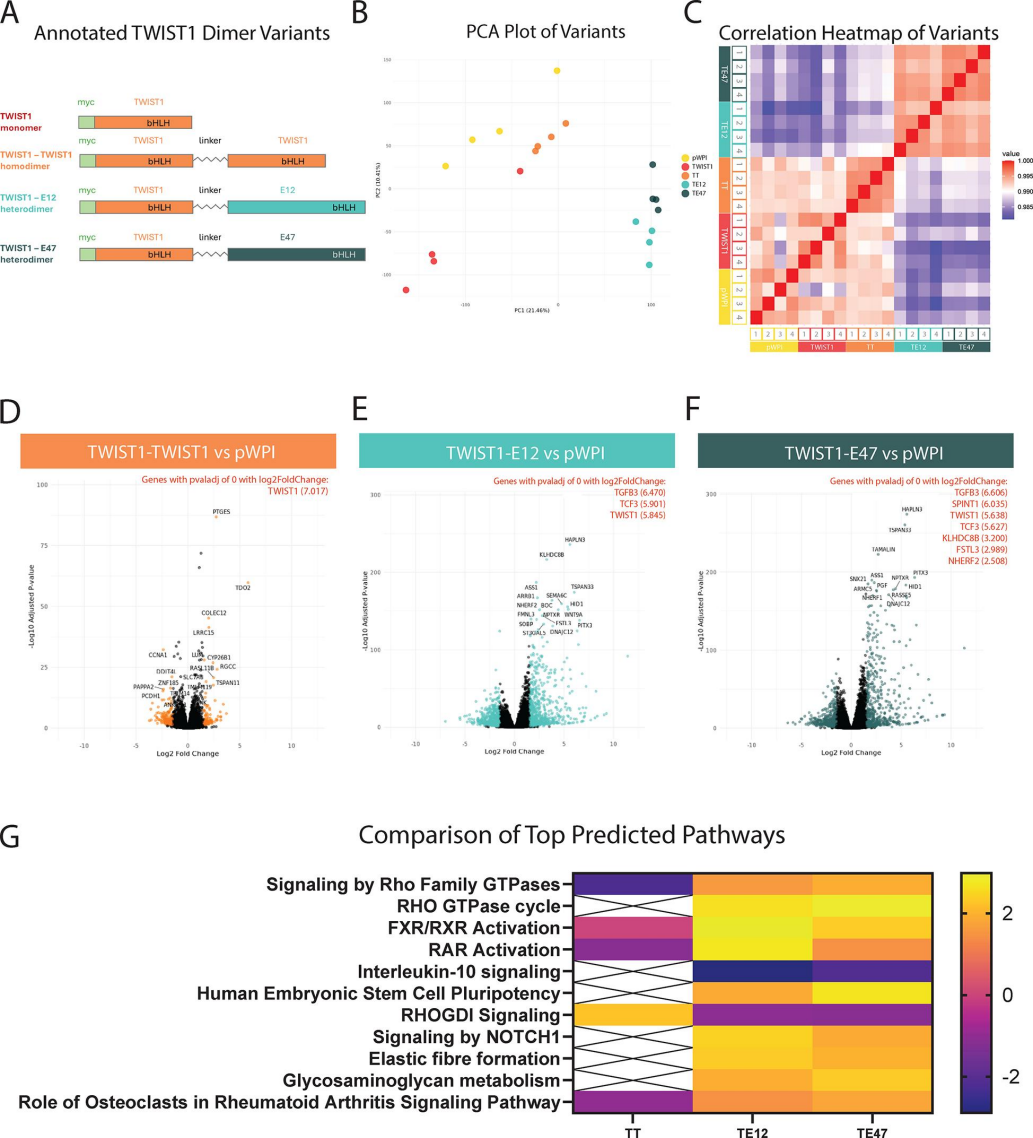
Transcriptional profiles of TWIST1 dimers. HCASMCs were transduced with overexpression constructs of TWIST1 in vector backbone pWPI, including a monomer and 3 different forced dimer constructs, joined by a flexible linker **(A,B)** Principal component analysis (PCA) was performed on the samples (*n* = 4 replicates per group) and visualized. **(C)** Heatmap of a Pearson Correlation (midpoint = 0.990) for all samples. **(D–F)** Volcano plots of DE gene expression for each dimer compared to the empty vector. Each comparison highlights in color significant DE genes above an adjusted *p*-value below 0.05 and a log2 fold change above an absolute value of 1.5. The top 20 genes are labeled in each plot, and highly significant genes (adjusted *p*-value of 0) are listed in red with their corresponding log2 fold change values. **(G)** Significant DE genes were used to identify Top Predicted Pathways across the dimers, visualized with a heatmap.

To understand the effect of TWIST1 dimerization on SMC phenotype, we generated overexpression constructs for the TWIST1 monomer, TWIST1-TWIST1 homodimer (TT), and TWIST1 heterodimers with TCF3-encoded E protein splice variants, TWIST1-E12 (TE12) and TWIST1-E47 (TE47) (Figure 1A). The dimer constructs were designed with a flexible linker to force TWIST1 binding with its respective dimer partners (14). Human coronary artery smooth muscle cells (HCASMCs) were serum starved for 24 h before lentiviral transduction with each of the constructs or empty vector control (pWPI). Following viral incubation for 48 h, RNA was isolated and bulk RNA-sequencing (RNA-Seq) was performed in replicates of 4. To validate overexpression of each dimer variant in the bulk RNA-seq analysis, the average Transcripts Per Million (TPM) of *TWIST1* in each condition was calculated. Empty vector (pWPI) samples (representing endogenous *TWIST1* expression) had an average TPM of 16.36 counts with overexpression constructs showing a 69- to 182-fold increase over this endogenous expression level. To understand the effect of each construct on HCASMC transcriptomes, Principal Component Analysis (PCA, Figure 1B) and Pearson's Correlation Analysis (Figure 1C) were performed to visualize the differences across the samples. These analyses revealed that the TWIST1 heterodimers TE12 and TE47 exhibit a distinct transcriptional signature from the TWIST1 monomer or the TT homodimer. The relative similarity between the TWIST1 monomer and the TT homodimer transcriptional profiles suggests that overexpression of the TWIST1 monomer in excess likely drives the homodimerization of TWIST1. Therefore, our analysis focused on analysis of the forced dimer constructs.

Differentially expressed gene (DEG) analysis was performed using DESeq2 for each dimer-treated sample compared to empty vector control. Significant DEGs were defined as those with an adjusted *p*-value less than 0.05 and a log2 fold change (log2FC) with an absolute value greater than 1.5 (Figures 1D–F). These volcano plots illustrate the differing effects of TWIST1's dimer partners on the regulation of its downstream targets, as each dimer resulted in the detection of varying numbers of significant DEGs. Compared to the empty vector control, the TT homodimer resulted in 274 significant DEGs (100 up; 174 down), the TE12 heterodimer resulted in 1276 significant DEGs (686 up; 590 down), and the TE47 heterodimer resulted in 1081 significant DEGs (586 up; 495 down). Overall, these data demonstrate that the heterodimers have a much broader effect on gene expression compared to that of the homodimer.

### 3.2 TWIST1 heterodimers activate key SMC signaling pathways to promote ECM production and organization

Comparison of top predicted pathways across dimers revealed often opposite regulation of key pathways by TT homodimers vs. TE12 or TE47 heterodimers. For example, heterodimers upregulated the related “Signaling by Rho Family GTPases” and “Rho GTPase Cycle” pathways (Figure 1G; Supplementary Figure S1A), while downregulating the corresponding inhibitory “RHOGDI Signaling” pathway. In contrast, TT homodimers regulated these same pathways in opposite directions. The Rho family GTPases are known to control cellular processes including cell adhesion, migration, and proliferation, and Rho-specific guanine nucleotide dissociation inhibitors (RHOGDIs) inhibit these GTPases by preventing dissociation of GDP (27). The comparison analysis across dimers shows upregulation of molecules involved in these pathways (ARHGAP27, ARHGAP45, ARHGAP9, ARHGEF3, BAIAP2, PIK3C2B, PIK3R6, RHOB, RHOV, SH3BP1, SH3PXD2A, SEPTIN3, SPETIN6) by heterodimers, further supporting their role in changes in HCASMC cellular adhesion, proliferation, and migration. Of note, TGFB3 was found to be one of the top DE genes, and can signal through Rho/ROCK to promote ECM production and organization (28, 29). Other key pathways upregulated by TE12 and TE47 heterodimers included “Elastic fibre formation” (upregulation of *BMP4, EMILIN2, FBLN1, FBLN5, MFAP4, TGFB3, VTN*), and “Glycosaminoglycan metabolism” (upregulation of *BCAN, FMOD, HAS3, LUM, PRELP, ST3GAL4, OGN*). TE12 and TE47 heterodimers also upregulated the retinoic acid receptor (RAR) pathway (Figure 1G; Supplementary Figure S1A), in contrast to TT homodimers, which downregulated this pathway.

Since TE12 and TE47 transcriptomes were highly similar, further analysis directly compared TWIST1 homodimer to the TE12 heterodimer to avoid redundancy. To further investigate the effect of TE12 on HCAMSC transcriptome compared to TT, significant DEGs between these dimers (Supplementary Figure S1B) were analyzed using Qiagen's Ingenuity Pathway Analysis (IPA) software. This analysis revealed that various top predicted pathways are upregulated by TE12 compared to TT (Supplementary Figure S1C) include “Molecular Mechanisms of Cancer” and “Wound Healing Signaling Pathway”. These pathways are enriched for aGPCRs (*ADGRE2, ADGRE3*), GPCRs (*GPR84, GPR162, GPR37L1*), integrin subunits (*ITGA3, ITGA7*), matrix metallopeptidases (*MMP8, MMP23*), and Ras homolog family members (*RHOB, RHOV*). This direct comparison between dimers further supports heterodimer-driven changes to HCASMC transcriptomics that directs cell-matrix interactions.

### 3.3 TWIST1 homodimers promotes SMC migration and proliferation

**Figure 2:**
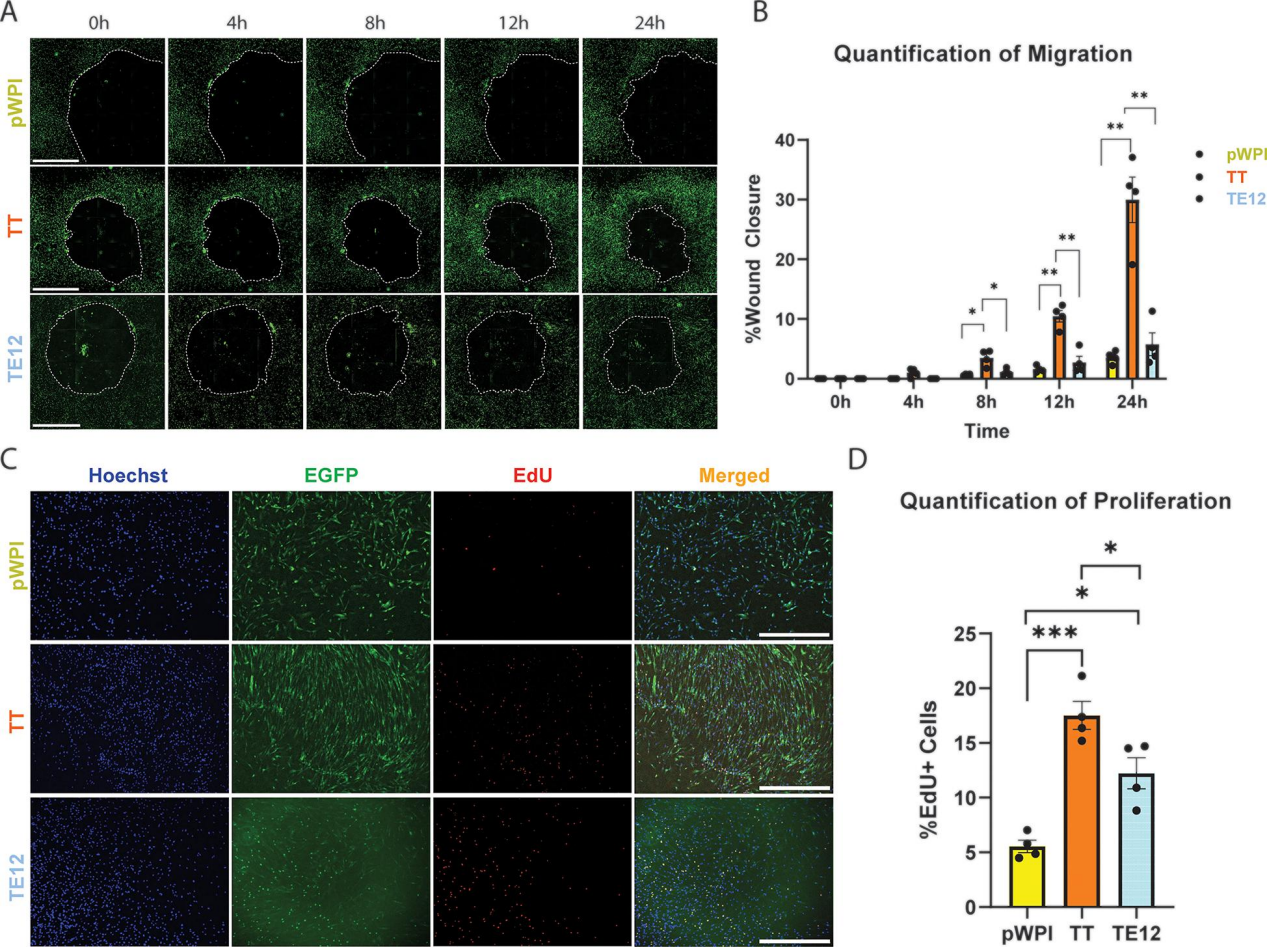
Functional analysis of HCASMCs transduced with TWIST1 dimers. **(A)** A wound healing assay was performed on HCASMCs transduced with either empty pWPI vector, the TWIST1-TWIST1 forced homodimer (TT), or the TWIST1-E12 heterodimer (TE12). Wound closure was observed at time points: 0 h, 4 h, 8 h, 12 h, and 24 h. The scale bar represents a length of 1 mm. **(B)** The wound area was measured at each time point, and quantified. Significant changes in wound closures were observed at 8 h, 12 h, and 24 h time points (*t*-test). **(C)** A baseclick EdU Assay was performed on these cells over 12 h. Cell nuclei were observed using Hoechst, transduced HCASMCs were identified via EGFP, and proliferating cells labeled with EdU produced a red signal. The scale bar represents a length of 1 mm. **(D)** Quantification of proliferation.

To investigate the corresponding functional effect of TWIST1 dimer composition on HCASMC proliferation and migration, cells were serum starved for 24 h, followed by transduction with lentivirus vectors containing the TT homodimer, the TE12 heterodimer or empty vector control. Antibody staining was performed using an anti-TWIST1 antibody to validate nuclear localization in transduced cells (Supplementary Figure S2A). After 48 h, a wound healing assay was performed. At time 0 hours, a circular wound was made, and the wells containing HCASMCs were imaged every 4 h up to 24 h (Figure 2A). Transduction efficiency was quantified by colocalizing EGFP and DAPI signal, and is represented as cells with colocalized signal divided by all DAPI-positive particles. We report no significant difference in the percentage of cells transduced between each dimer variant, with approximately 90% of HCAMSCs transduced across all dimer variants. (93.49% of pWPI; 88.62% of TWIST1-E12; 93.65% of TWIST1-TWIST1) (Supplementary Figure 2B). To quantify migration of the HCASMCs, the percent wound closure was calculated for each time point: ((AreaT0−AreaTt)/AreaT0)*100. Statistical analysis revealed that after only 8 h TT transfected cells were significantly more migratory than empty vector (pval 0.0243) and TE12 (pval 0.0307). After 12 h this effect was enhanced, with TT being significantly more migratory than empty vector (pval 0.0017) and TE12 (pval 0.0014). This remained consistent after 24 h for empty vector (pval 0.0057) and TE12 (pval 0.0036) (Figure 2B). There was no significant difference detected between TE12 and empty vector at any time point. At the 24-hours post-wound timepoint, the non-transduced (EGFP-) cells that had migrated were quantified as a percentage of all cells that had migrated, and we found no significant difference in the number of non-transduced cells within the wound-closure area (Supplementary Figure S2C).

We then performed proliferation assays using the baseclick EdU system in HCASMCs to capture cell proliferation over a 12 h incubation period. Proliferating cells were identified by co-localization of nuclei marker Hoechst, EGFP (to identify transduced cells), and the EdU signal. (Figure 2C). The percentage of EdU + cells was calculated by dividing the number of HCASMCs with triple-colocalized signal by the total number of cells containing both EGFP and Hoechst. Quantification of signal revealed that TT-transduced cells were significantly more proliferative than empty vector (pval 0.0009) and TE12-transduced cells (pval 0.0337). TE12-transduced cells were also significantly more proliferative than empty vector (pval 0.0128), but to a lesser degree. Together, functional analysis of the TWIST1 dimers suggest that TT substantially increases HCASMC migration and proliferation, while TE12 modestly increases proliferation but not migration. Proliferation was also quantified for DAPI + EdU + EGFP- cells to understand the contribution of non-transduced cells to total proliferation (Supplementary Figure S2D). The non-transduced cells are fundamentally different from their transduced counterparts, with very low rates of proliferation compared to the transduced HCAMSCs. Ultimately, these cells contribute minimally to observed proliferation, representing 1.79% of pWPI-treated cells, 0.50% of TWIST1-TWIST1-treated cells, and 0.41% of TWIST1-E12-treated cells. Interestingly, although TT resulted in fewer DEGs, it exhibited a more pronounced effect on HCASMC migration and proliferation. In contrast, while TE12 resulted in more DEGs, this dimer appears to have a greater effect on ECM production/organization than on proliferation and migration. Together, these data suggest that the TWIST1 homodimer and heterodimers have distinct effects on SMC phenotype, independently regulating various aspects of SMC phenotype.

### 3.4 The C-terminus is a major determinant of TWIST1's transcriptional activity in SMCs

**Figure 3:**
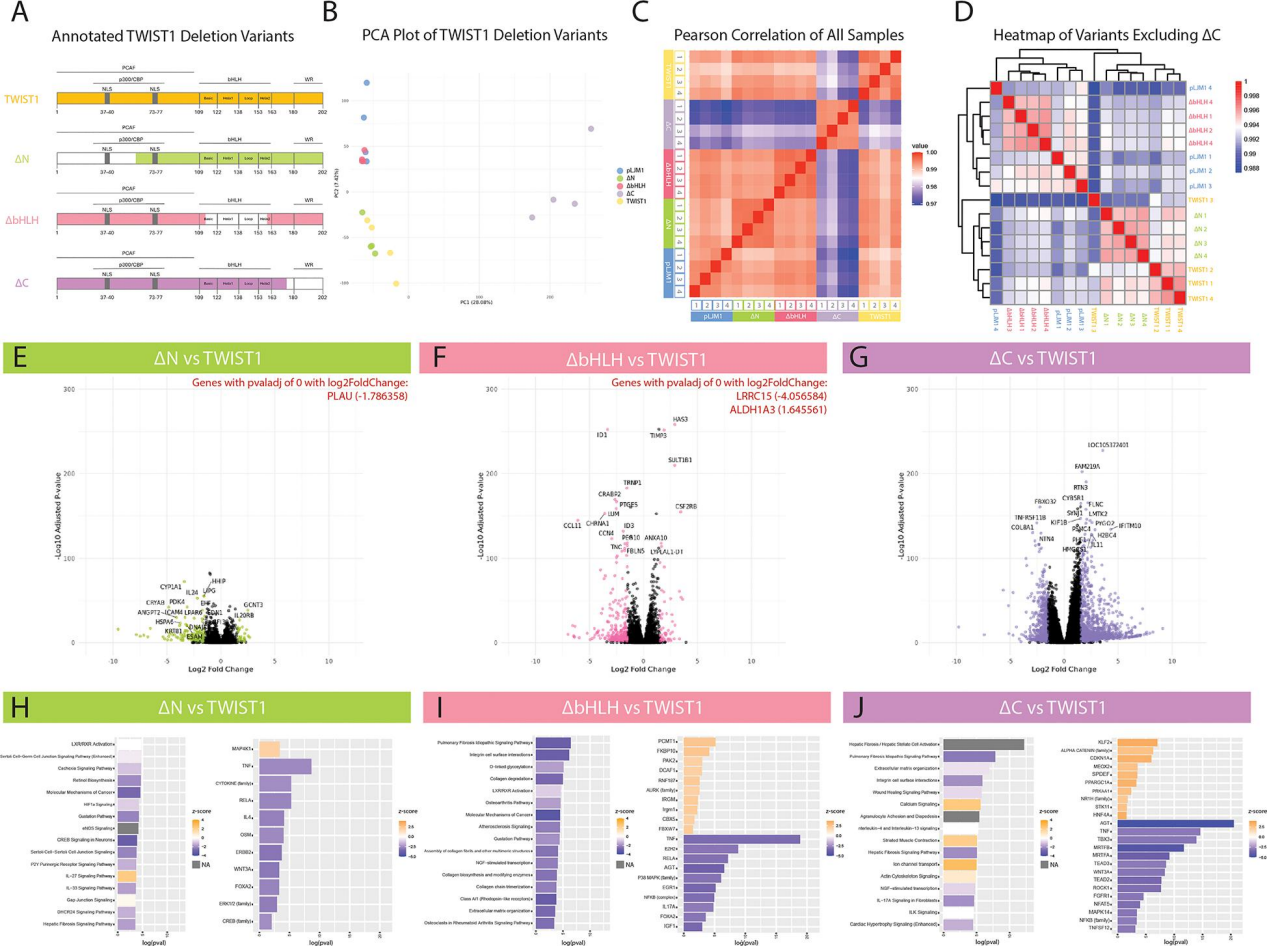
Transcriptional investigation of the role of TWIST1 domains. **(A)** HCASMCs were transduced with overexpression constructs containing full-length TWIST1, TWIST1 deletion variants, or empty pLJM1 vector. White-space in each construct represents the region deleted from TWIST1. **(B)** Principal component analysis (PCA) was performed on the samples (*n* = 4 replicates per group). **(C)** Heatmap of Pearson Correlation (midpoint = 0.985) for all samples. **(D)** Additional heatmap visualization excluding ΔC, using hierarchical clustering. (**E–G**) Volcano plots of DE genes for each variant compared to full-length TWIST1. Each comparison highlights in color significant DE genes above an adjusted *p*-value below 0.05 and a log2 fold change above an absolute value of 1.5. The top 20 genes are labeled in each plot, and highly significant genes (adjusted *p*-value of 0) are listed in red with their corresponding log2 fold change values. (**H–J**) Significant DE genes were used to identify Top Predicted Pathways (left) and Predicted Upstream Regulators (right) for each construct compared to full-length TWIST1. Bar plots are displayed with upregulated pathways/molecules colored orange and downregulated pathways/molecules colored blue. Grey bars represent pathways with no calculated z-score.

To determine the function of different TWIST1 domains on SMC phenotype, we generated TWIST1 mutants that lacked each of three primary domains of TWIST1: 1) the N-terminus (ΔN); 2) the basic helix-loop-helix domain (ΔbHLH); and 3) the C-terminus (ΔC) (Figure 3A). As above, HCASMCs were transduced with either empty vector, full-length TWIST1, or one of the domain deletion variants and prepared for bulk RNA-Seq. As performed with the dimer variants, the average Transcripts Per Million (TPM) of *TWIST1* in each condition was calculated to validate overexpression. Empty vector (pLJM1) samples (representing endogenous *TWIST1* expression) had an average TPM of 43.59 counts, with overexpression constructs showing a 209- to 2,129-fold increase over this endogenous expression level. PCA analysis (Figure 3B) revealed that full-length TWIST1 and the ΔN variant showed similar transcriptional profiles, while the ΔbHLH variant was superimposable with the empty vector control. In contrast, the ΔC variant displayed a markedly different transcriptional profile in both PCA and Pearson's Correlation analyses (Figures 3B,C). As this large transcriptional shift overshadowed differences between other deletion variants, we also performed hierarchical clustering by Euclidean distance for all samples excluding the ΔC variant (Figure 3D). This analysis confirmed that the ΔN variant displayed a similar transcriptomic profile to full-length TWIST1, and again showed the similarity between the ΔbHLH variant and empty vector. Given both the PCA and Pearson analyses, we conclude that loss of the bHLH domain renders TWIST1 transcriptionally non-functional, while loss of the N-terminus has a relatively minor effect on TWIST1's transcriptional activity in SMCs.

To further interpret the effect of each variant, DEG analysis was performed comparing ΔN, ΔbHLH, and ΔC to full-length TWIST1. Each comparison yielded a different number of DEGs (Figures 3E–G). As visualized by the volcano plot (Figure 3G), ΔC results in the highest number of DEGs compared to TWIST1, indicating a unique role for the C-terminus in regulating TWIST1's downstream targets. For ΔN and ΔbHLH, significant DEGs were defined as having an adjusted *p*-value below 0.05 and an absolute value for log2FC greater than 1.5. However, using these same parameters, ΔC had 1494 DEGs. Therefore, an adjusted *p*-value below 10^−10^ was used for the ΔC variant, which reduced the inclusion of lowly-expressed genes and allowed for a more stable analysis. Compared to full-length TWIST1, ΔN resulted in 197 significant DEGs (27 up; 170 down), ΔbHLH resulted in 381 significant DEGs (59 up; 322 down) and, using the more stringent cutoffs for significance, ΔC resulted in 935 DEGs (564 up; 371 down). Notably, ΔC results in the net upregulation of genes, suggesting that this domain acts on balance as an inhibitor.

The significant DEG lists comparing each deletion variant to full-length TWIST were analyzed using IPA to identify top predicted pathways and upstream regulators (Figures 3H–J). Consistent with the PCA and Pearson correlation analyses showing minimal deviation from full-length TWIST1, DEGs from the ΔN variant displayed only relatively modest pathway enrichment in this analysis, including “LXR/RXR Activation” (*CLU, IL1RAPL1, IL1RL1, NGFR, SAA1, SAA2*) and “Retinol biosynthesis” (*AADAC, CES1, DHRS9, LIPG, LRAT*).

The ΔbHLH variant displayed downregulation of various collagen (C*OL10A1, COL5A3, COL7A1, COL8A1, COL9A2)*, integrin (*ITGA7*), matrix metalloprotease (*MMP3, MMP12, MMP24*), ADAM metalloproteinase (*ADAMTS9, ADAMTS14, ADAMTS15*), mucin (*MUC19, MUC20*), WNT (*WNT7B, WNT9A*), and other ECM-protein genes (*LUM, TNC, ICAM4*) resulting in top predicted pathways including “Pulmonary Fibrosis Idiopathic Signaling Pathway”, “Integrin cell surface interactions”, “O-linked glycosylation”, “Collagen degradation”, “Assembly of collagen fibrils”, “Collagen trimerization”, and “ECM organization” (Figure 3I). Furthermore, “Atherosclerosis Signaling” was among the top pathways predicted to be downregulated with ΔbHLH via downregulation of relevant genes *APOD, CCL11, CD36, CLU, COL10A1, COL5A3, IL1RN, MMP5, and MSR1*. Given the ΔbHLH variant is essentially non-functional, this comparison suggests that full-length TWIST1 is heavily-involved in modulating the ECM in SMCs.

### 3.5 The C-terminus is necessary for TWIST1 to modulate Rho/ROCK signaling and ECM production

The ΔC variant also resulted in the downregulation of similar ECM-related pathways, including “Pulmonary fibrosis idiopathic signaling pathway”, “Extracellular matrix organization”, “Integrin cell surface interactions, and “Wound healing signaling pathway”. However, the predicted regulators mediating these effects were significantly different, and included the angiotensin pathway (AGT), the transcription factors MRTFA, MRTFB, TEAD2, TEAD3, and the kinase ROCK1 (Figure 3J). Importantly, AGT signals through RhoA/ROCK1, which leads to actin polymerization and MRTFA/MRTFB nuclear localization. Similarly, activation of RhoA/ROCK1 also leads to YAP/TAZ nuclear localization and cooperation with TEADs. The identification of multiple pathways that converge on Rho/ROCK signaling strongly implicates that TWIST1 interacts with this core pathway via its C-terminal domain.

Interestingly, while the ΔN and ΔbHLH variants largely resulted in downregulation of genes and predicted pathways, the ΔC variant showed predicted upregulation of pathways including “Calcium Signaling”, “Striated Muscle Contraction”, “Ion channel transport”, and “Actin-Cytoskeleton Signaling” (Figure 3J). Predicted upregulation of these top pathways is driven by upregulation of myosin heavy chains (*MYH1, MYH2, MYH3, MYH4, MYH8, MYH14*), myosin 1A (*MYO1A*), myosin binding proteins (*MYBPC1, MYBPC2*), titin-cap (*TCAP*), titin (*TTN*), tropomyosin 1 (*TPM*), anoctamins (*ANO1, ANO2*), bestrophin 3 (*BEST3*), ATPase sarcoplasmic/endoplasmic reticulum calcium transporting 1 (*ATP2A1*), ATPase hydrogen transporting subunits (*ATP6V0A1, ATP6V0A4, ATP6V1B2*), integrin subunits (*ITGA7, ITGAM, ITGAX*), calcium voltage-gated channel subunits (*CACNA1B, CACNA1E*), mitochondrial calcium uptake 1 (*MICU1*), and ryanodine receptor (*RYR1, RYR2*) genes by ΔC compared to TWIST1. Most of these genes are expressed specifically in skeletal myocytes and not smooth muscle cells, and were predicted to be regulated by MEF2C (data not shown). Given the role of the C-terminus of TWIST1 in suppressing MEF2 activity (19), it is possible that deletion of the C-terminus results in de-repression of MEF2 and unmasking of a latent skeletal myocyte gene expression program in SMCs.

### 3.6 SMC phenotypic modulation and migration/proliferation are independently regulated by different domains of TWIST1

To further understand the effect of different TWIST1 domains on migration, the wound healing assay was performed on HCASMCs transduced with full-length TWIST1, ΔN, ΔbHLH, ΔC or empty vector. Antibody staining was performed using an anti-MYC antibody to validate nuclear localization in transduced cells (Supplementary Figure S3A). As performed with the dimer variants, transduction efficiency was quantified by colocalizing EGFP and DAPI signal, and is represented as cells with colocalized signal divided by all DAPI-positive particles (Supplementary Figure S3B). We report no significant difference in the percentage of cells transduced between deletion variants, with approximately 95% of HCAMSCs transduced across all deletion variants (97.99% of pLJM1; 95.19% of TWIST1; 93.59% of ΔN, 95.24% of ΔbHLH; 93.05% of ΔC).

**Figure 4:**
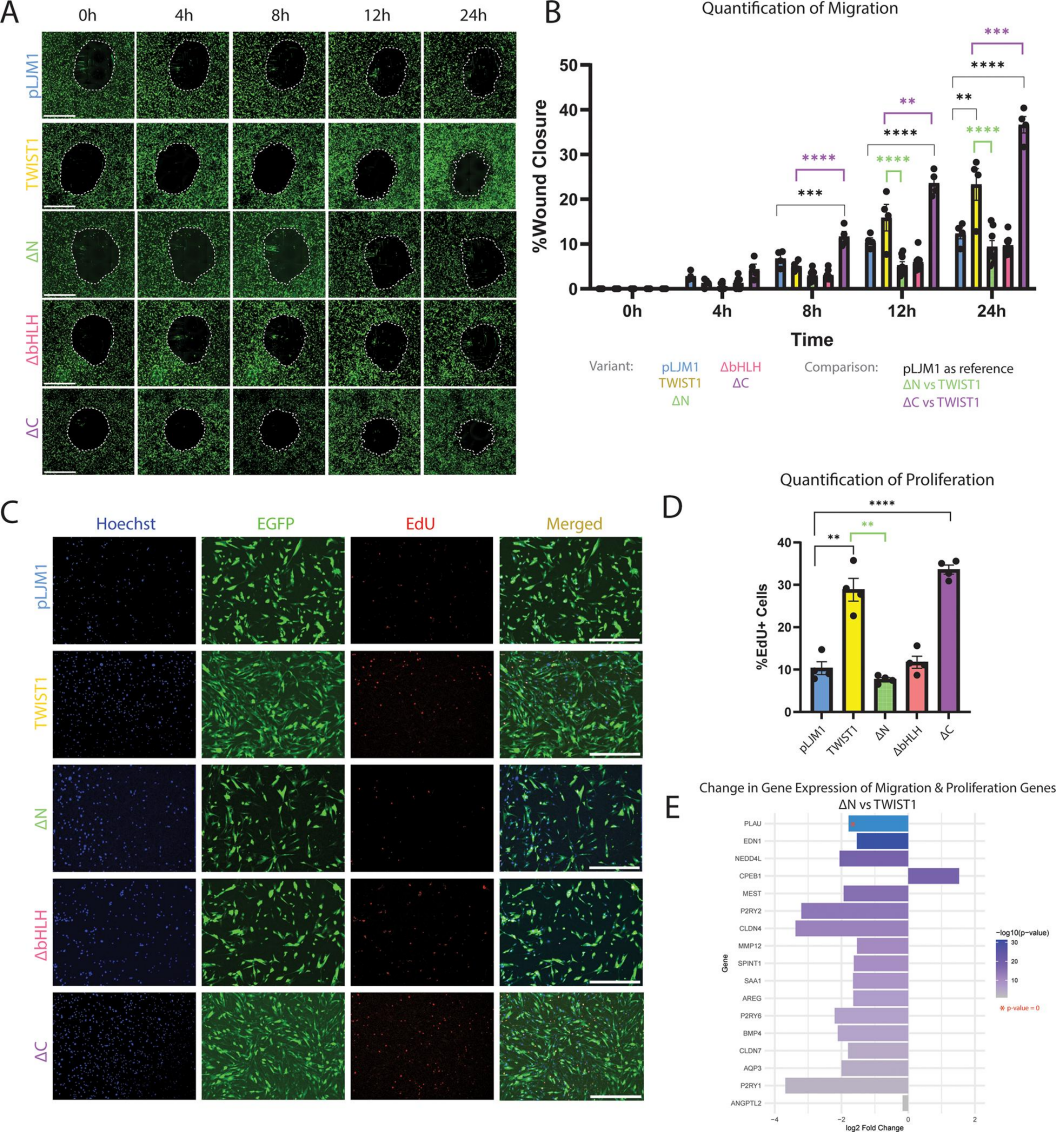
Functional analysis of HCASMCs transduced with TWIST1 variants. **(A)** A wound healing assay was performed on HCASMCs transduced with either empty pLJM1 backbone, full-length TWIST1, or the TWIST1-variants (ΔN, ΔbHLH, ΔC). Wound closure was observed at time points: 0 h, 4 h, 8 h, 12 h, and 24 h. The scale bar represents a length of 1 mm. **(B)** The wound area was measured at each time point, and quantified, and significance was determined using one-way ANOVA. **(C)** A baseclick EdU Assay was performed on these cells over 12 h. Cell nuclei were observed using Hoechst, transduced HCASMCs were identified by EGFP, and proliferating cells labeled with EdU produced a red signal. The scale bar represents a length of 1 mm. **(D)** Percentage of proliferating cells were quantified. **(E)** Genes associated with migration and/or proliferation were visualized for log2 fold change between the ΔN variant compared to full-length TWIST1. The genes are ordered by -log(*p*-value), and PLAU has a *p*-value of 0.

As before, the wound area was measured every 4 h up to 24 h after wound formation (Figure 4A). Quantification of this migration assay (Figure 4B) showed that, in comparison to empty vector, full-length TWIST1 significantly increased migration after 24 h (pval 0.0048). In contrast, deletion of the N-terminal domain in ΔN completely abrogated TWIST1's ability to promote migration after 12 hours (pval <0.0001) and 24 h (pval <0.0001). Similarly, deletion of the bHLH domain in ΔbHLH resulted in the inability to promote migration after 24 h (pval <0.0001). In contrast, ΔC retained the ability to promote migration, being significantly more migratory after 8 h (pval 0.0008), 12 h (pval <0.0001), and 24 h (pval <0.0001) than the empty vector. ΔC was also significantly more migratory than full-length TWIST1 after 8 h (pval <0.0001), 12 h (pval 0.0090), and 24 h (pval 0.0006). Again, 24 h after wound creation, the non-transduced (EGFP-) cells that had migrated were quantified as a percentage of all cells that had migrated, and we found no significant difference in the number of non-transduced cells within the wound-closure area (Supplementary Figure S3C).

In addition to assessment of the effect of TWIST1 variants on migration, the EdU Assay was performed to evaluate the effect on proliferation for an incubation period of 12 h. Proliferating HCASMCs were again identified by colocalizing Hoechst, EGFP, and EdU signals (Figure 4C). Quantification of the proportion of proliferating transduced cells revealed that full-length TWIST1 significantly increases proliferation compared to the empty vector (pval 0.0023). Importantly, deletion of the N-terminal domain in ΔN completely abolishes TWIST1's ability to promote proliferation (pval 0.0037). Similarly, deletion of the bHLH domain in ΔbHLH variant resulted in the loss of TWIST1's ability to promote proliferation (pval 0.0033). However, the ΔC variant maintained the ability to promote proliferation compared to empty vector (pval <0.0001), indicating that this domain is not essential for TWIST1's ability to promote proliferation in HCASMCs (Figure 4D). Proliferation was also quantified for DAPI + EdU + EGFP- cells (Supplementary Figure S3D), contribute minimally to observed proliferation, representing 0.06% of pLJM1-treated cells, 1.10% of TWIST1-treated cells, 2.71% of ΔN-treated cells, 4.14% of ΔbHLH-treated cells, and 3.08% of ΔC-treated cells.

Unlike deletion of the bHLH domain, which abolished TWIST1's effect on the SMC transcriptome (Figures 3B,D), loss of the N-terminus completely inhibited TWIST1's ability to induce proliferation and migration despite a minimal change in the transcriptome. Comparing cell function pathways between full-length TWIST1 and the ΔN variant revealed significant predicted downregulation of cell movement/migration (data not shown). The top 10 migration/proliferation pathways in this analysis were analyzed to identify common genes across multiple terms, which are shown in Figure 4E. Although the TWIST1-TWIST1 dimer variant pathway analysis did not detect a signal for migration or proliferation, it resulted in a significant increase in proliferation and migration in the functional assays. Therefore, top proliferation and migration pathways for the heterodimer and homodimer were analyzed to identify common genes across top pathways. Several genes were identified and log2FoldChange values were analyzed across the TWIST1-TWIST1 vs. pWPI, TWIST1-E12 vs. pWPI, and ΔN vs. TWIST1 comparisons (Supplementary Figure S3E). The corresponding *p*-values can be found in Supplementary Table S3. Our analysis reveals that several genes upregulated by the dimer variants are downregulated with ΔN (*SEMA6C, PTGES, ABCB1, CCL7, BMP4, MMP12*), providing insight into specific genes targeted by TWIST1 to promote proliferation and migration that are lost with deletion of the N-terminal domain.

### 3.7 Deletion of TWIST1 C-terminus is predicted to inhibit SMC phenotypic modulation during disease

**Figure 5:**
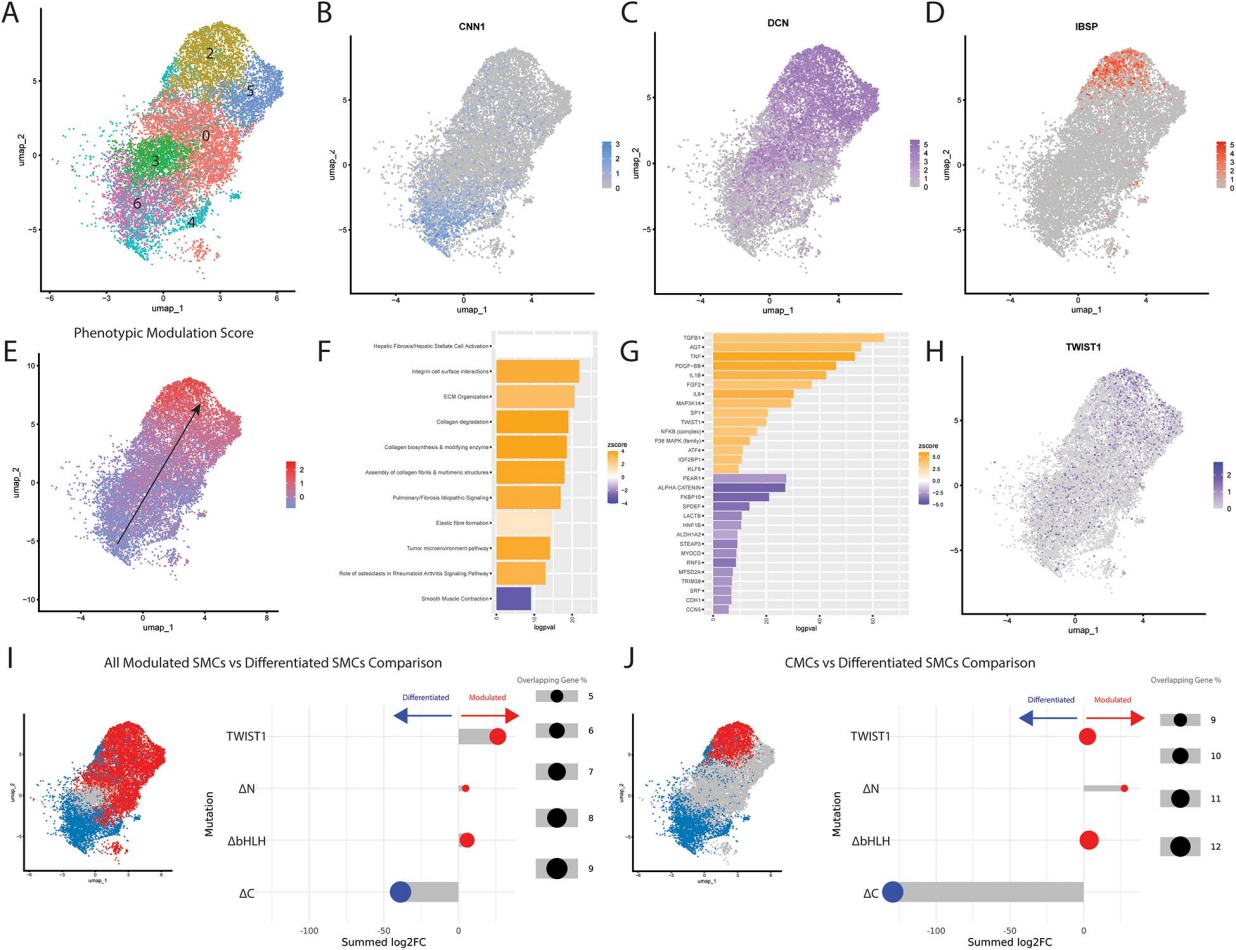
Relevance of TWIST1 domains in SMC phenotypic modulation from human endarterectomy data. **(A)** UMAP of SMCs from combined human endarterectomy samples from 20 human patients, clustered. Cluster 1 does not exist for this UMAP. Feature plots of differentiated SMC marker *CNN1* **(B)**, modulated FMC marker *DCN* **(C)**, and CMC marker *IBSP* **(D)** illustrate the transcriptional transition of SMCs. Top DE genes between phenotypically modulated and differentiated clusters were used to determine a Phenotypic Modulation Score for all SMCs, with higher scores indicating more phenotypic modulation **(E)** Significant DE genes were used to identify Top Predicted Pathways **(F)** and Top Predicted Upstream Regulators **(G)** Bar plots are displayed with upregulated pathways/molecules colored orange and downregulated pathways/molecules colored blue. **(H)** *TWIST1* expression increases during SMC phenotypic modulation. (**I,J**) The log2 fold change values of DE genes between each TWIST1 variant and empty vector control were cross-referenced with genes defining the *in vivo* transition between differentiated SMCs and all modulated SMCS **(I)** or between differentiated SMCs and CMCs **(J)**. These *in vitro* log2 fold change values were assembled into a score that represents whether the TWIST1 variant changes these genes in a manner consistent with decreased SMC modulation (blue) vs. increased SMC modulation (red).

To understand the relevance of TWIST1 in human disease, we downloaded single cell RNA-Sequencing (scRNA-Seq) data of human endarterectomy samples collected from 20 patients (26). Cells from all samples were integrated and SMC clusters were isolated based on expression of SMC markers (Figure 5A). These cells displayed a continuum of gene expression, including fully-differentiated SMCs (Figure 5A, clusters 4 and 6) expressing the highly-specific SMC marker *CNN1* (Figure 5B), cells that we have previously termed fibromyocytes (FMCs, Figure 5A, clusters 0, 3 and 5) that had lost SMC differentiation markers and gained markers such as *DCN* (Figure 5C), and cells that we have previously termed chondromyocytes (CMCs, Figure 5A, cluster 2) that express markers of osteoblasts/chondrocytes such as *IBSP* (Figure 5D). The top 7 DE genes between CMCs and differentiated SMCs (*IBSP, TMEM119, VCAM1, DCN, LUM, MXRA5,* and *PDPN*) were used to construct a ’SMC phenotypic modulation score’, which was used to visualize the degree of phenotypic modulation present for each cell (Figure 5E). Interestingly, the expression of *TWIST1* increased as cells displayed increasing SMC phenotypic modulation scores (Figure 5F). To understand the gene program that promotes phenotypic modulation (black arrow in Figure 5E), DE gene analysis was performed comparing cluster 2 (CMCs) to clusters 4 and 6 (SMCs)*.* This resulted in the identification of 2763 DEGs with an adjusted *p*-value below 0.05. These DEGs were analyzed using IPA and top predicted pathways (Figure 5C) and upstream regulators (Figure 5D) were identified. The top predicted pathways included upregulation of pathways including ECM organization, collagen-related pathways, and elastic fibre formation, and downregulation of smooth muscle contraction. Importantly, TWIST1 was identified among the top predicted upstream regulators of this phenotypic transition, in addition to molecules predicted to be downregulated with the TWIST1 deletion variants, including AGT, TNF, and NFKB. Additionally, master regulators of SMC differentiation, SRF and MYOCD, are among the top predicted downregulated upstream regulators.

To understand the relevance of our *in vitro* genetic manipulations to this process, we compared gene expression changes in the human carotid scRNAseq data to the changes in bulk RNAseq gene expression induced by our TWIST1 variants *in vitro*. The log2FC for DEGs comparing each TWIST1 variant to empty vector were summed into a score, based upon whether they increased or decreased with SMC phenotypic modulation *in vivo* (see Methods and Supplementary Table S1). DEGs for each variant were scored based upon their alignment with gene expression changes occurring between differentiated SMCs and i) all modulated SMCs (blue vs. red, Figure 5I, left panel) or ii) CMCs specifically (blue vs. red, Figure 5J, left panel). This revealed that, while TWIST1 modestly increased genes that were upregulated with SMC phenotypic modulation *in vivo*, the ΔC variant resulted in a gene expression profile that moved markedly toward a less modulated SMC phenotype (Figure 5I). This shift was further accentuated when compared to genes defining CMCs vs. SMCs (Figure 5J). In both cases, the transcriptional shift observed for the ΔC variant was predominantly driven by downregulation of genes expressed in modulated SMCs, rather than upregulation of genes expressed by differentiated SMCs (Supplementary Table S1). The same analysis was performed for each forced TWIST1 dimer compared to the empty vector (Supplementary Figures S4A,B). Finally, qPCR validation was performed to validate the effects of full-length TWIST1 on markers of SMC phenotype (Supplementary Figure S4C). These data reveal that TWIST1 overexpression significantly reduces expression of *TAGLN* (pval: 0.0220), but does not significantly affect other SMC markers such as *CNN1* or *ACTA2*. In contrast, TWIST1 overexpression resulted in significantly increased expression of modulated SMC genes including *DCN* (pval: 0.0087), *TNFRSF11B* (pval: 0.0385), *TMEM119* (pval: 0.0249), and *IBSP* (pval: 0.0286).

## 4 Discussion

TWIST1 dimer composition is known to influence its effects—for instance, elegant work in TWIST1 +/- mice modeling SCS, suggests that TWIST1 haploinsufficiency results in a reduction of TWIST1-E12 heterodimers, resulting in premature osteoblast differentiation and suture closure. Another study in embryonic stem cells suggested TWIST1-E12 promotes differentiation to mesenchyme and neural crest lineages, while TWIST1-TWIST1 maintained cells in a progenitor state. As the effects of TWIST1 dimer composition likely differ depending on the cell type and context, and given the recently recognized role of TWIST1 in modulating risk for vascular disease, we sought to determine the effect of dimer composition in SMCs. Further, almost nothing is known regarding the function of discrete TWIST1 domains in shaping SMC phenotype.

Our study reveals that TWIST1's effects on SMC phenotype are strongly dependent on its dimer composition (13). Specifically, we found that the TWIST1-TWIST1 homodimer promotes SMC proliferation and migration, whereas the TWIST1-E12 heterodimer instead enhances extracellular matrix (ECM) production and organization. These divergent effects suggest that TWIST1 dimers play specialized roles in modulating distinct aspects of SMC phenotype. Interestingly, the TWIST1-TWIST1 homodimer induced only modest changes in gene expression, yet it strongly enhanced SMC proliferation and migration. Similarly, deletion of the N-terminal region of TWIST1 abolished its effects on proliferation and migration without significantly altering the SMC transcriptome. These findings support a model in which the TWIST1 homodimer governs SMC behavior through a relatively focused transcriptional program, requiring its N-terminal domain for function. An annotated list of the DEGs identified for each comparison can be found in Supplementary Table S4.

In contrast, the TWIST1-E12 heterodimer induced broader transcriptional changes, particularly those involved in ECM remodeling and Rho/ROCK signaling pathways. While it had a modest effect on proliferation and no effect on migration, its transcriptional signature suggested a role in promoting SMC de-differentiation. This role appears to be mediated by the C-terminal domain of TWIST1, as deletion of this region resulted in transcriptional shifts antagonistic to those induced by the TWIST1-E12 heterodimer, including inhibition of angiotensin and Rho/ROCK signaling and impaired ECM gene expression. The large effect of the C-terminal deletion suggests that this TWIST1 variant functions in a dominant-negative manner, in contrast to the bHLH domain deletion which simply rendered TWIST1 non-functional with respect to all parameters tested.

This unexpected and completely novel link between TWIST1 and angiotensin-Rho/ROCK signaling is intriguing given that the 7p21.1 locus that modulates *TWIST1* expression is also highly associated with hypertension in GWAS. Specifically, genetically-mediated increases in *TWIST1* expression are associated with increased risk of hypertension (30, 31). Our present findings are directionally consistent with these observations, because the dominant-negative ΔC TWIST1 variant is predicted to result in reduced angiotensin and Rho/ROCK signaling in SMCs. The importance of this connection is underscored by the finding that SMC-specific deletion of GRAF3 (32), an inhibitory RhoGAP and causal GWAS gene for hypertension, resulted in hypertension and an increased response to angiotensin infusion in mice. The convergence of two hypertension GWAS genes on a common pathway underscores the importance of this pathway and of understanding the precise mechanism by which TWIST1 modulates it.

Previous work showing increased SMC phenotypic switching *in vivo* and *in vitro* with TWIST1 protein overexpression has suggested reduction of microRNA-143/145 and p68 repression, which regulate SMC differentiation markers, as potential mechanisms (33). However, this study also reported no direct interplay between TWIST1 and p68 (33). In the context of atherosclerosis, our data do not support previously reported findings that TWIST1 suppresses expression of traditional SMC differentiation markers. Instead, TWIST1 appeared to upregulate a set of modulated SMC markers, many of which are ECM-related. This suggests that the previously observed “de-differentiation” induced by TWIST1 may not reflect a loss of SMC identity *per se*, but rather a shift toward an ECM-remodeling, synthetic phenotype. Stated another way, the TWIST1-E12 heterodimer appears to promote a less differentiated SMC state, consistent with previous findings during development that TWIST1 promotes a multipotent mesenchymal state by inhibiting differentiation into myogenic or osteogenic lineages (13–15, 19). This transition is also reported to involve increased collagen synthesis (36). Recent studies find that second heart field (SHF)-derived SMCs possess a distinct transcriptional profile from that of cardiac neural crest (CNC)-derived SMCs, in which SHF-derived SMCs overexpress collagen synthetic genes (34, 35). Further analysis revealed that SHF-derived SMCs shows enriched chromatin accessibility for Twist1 binding motifs, supporting a lineage-specific role for Twist1 in regulating SMC collagen synthesis (34, 35). Furthermore, our data demonstrate that TWIST1's effects on proliferation/migration are separable from its effects on phenotypic modulation. These functions are controlled by distinct TWIST1 domains and dimer partners, highlighting that SMC de-differentiation and proliferation/migration are not obligatorily linked, but are independently regulated processes.

We unexpectedly found that, despite only modest transcriptomic differences compared to wild-type TWIST1, the ΔN variant was completely unable to induce proliferation or migration in HCASMCs, but appeared to have minimal effect on SMC differentiation. In contrast, the ΔC variant primarily affected the SMC transcriptome and SMC modulation. Together, our data indicate that distinct domains of TWIST1 regulate different aspects of SMC biology. Further, although SMC proliferation and migration often occur simultaneously with de-differentiation, it appears that these processes are not always coupled and likely result from distinct regulatory programs.

Interestingly, deletion of the C-terminal domain resulted in upregulation of skeletal muscle–related genes involved in calcium signaling, ion transport, and contraction, which are predicted to be regulated by MEF2C. While these genes are expressed at low levels in SMCs and are unlikely to directly contribute to SMC phenotype, the involvement of MEF2C is notable because TWIST1 has indeed been shown to inhibit MEF2 activity in skeletal myocytes through its C-terminal domain (19), likely via interaction as a TWIST1-E12 heterodimer. Because the role of the MEF2 family in SMCs is not well-characterized, it is possible that one or more MEF2 genes also regulate additional gene programs relevant to SMC plasticity, which warrants further investigation. Assays such as chromatin immunoprecipitation sequencing (ChIP-Seq) could be used in the future to map the binding landscape of *TWIST1* variants to gain more in-depth mechanistic insight on its interactions with other transcription factors such as MEF2C. Such insights would clarify how TWIST1's structural integrity influences the downstream transcriptional programs it regulates and which co-factors are necessary for its mechanisms.

In our study, deletion of the bHLH domain resulted in a non-functional TWIST1. However, the bHLH domain is critical for both DNA-binding and dimerization, and a limitation of our current model is ablation of this entire domain. Therefore, future studies will need to dissect the role of specific regions within this domain in SMCs. It is also worth noting that while this *in vitro* study was performed in the absence of proinflammatory cytokines and other proatherogenic stimuli that are present in the atherosclerotic environment, our overlapping analysis with the human coronary endarterectomy data supports that *TWIST1* overexpression is relevant in the disease context. Future work using *in vivo* models of atherosclerosis with SMC-specific *TWIST1* overexpression or knockdown will be important for better understanding how *TWIST1* alters expression of its downstream targets and contributes to lesion burden in the complex atherosclerosis environment.

Overall, our study establishes that TWIST1 exerts complex, domain-specific, and dimer-dependent effects on SMC phenotype. These findings provide mechanistic insight into TWIST1 function in adult vasculature and implicate new pathways through which TWIST1 may modify risk for multiple human vascular diseases.

## Data Availability

The original contributions presented in the study are publicly available. This data can be found under ArrayExpress accession E-MTAB-15722 (https://www.ebi.ac.uk/biostudies/ArrayExpress/studies/E-MTAB-15722).
