# Supplementary material for: Functional analysis of TWIST1 domains regulating smooth muscle cell phenotype
Source: Front Cardiovasc Med. 2025 Oct 31;12:1659847. doi: 10.3389/fcvm.2025.1659847 (PMC12615416; doi:10.3389/fcvm.2025.1659847)
Supplement: Supplementary file 2 [file Table2.docx]

**Supplemental Table 2: Table of Primers**

| Primer | Sequence |
| --- | --- |
| Twist1-T2A-Fwd | 5’ CTGGTTTAGTGAACCGTCAGATCCGCTAGCATGATGCAGCACGTGTCCAGC 3’ |
| Twist1-T2A-Rev | 5’ AGCAGTGATCCGCGTCCCTGGTGGGACGCGGACATGGA 3’ |
| ΔN-Fwd | 5’TGAACCGTCAGATCCGCTAGATGGAGCAGAAACTCATCTCAGAAGAGGATCTG GGAGGCGGCGACG 3’ |
| ΔbHLH-N-Rev | 5’ GCGACTGGGTGCCGCCTCCGACGC 3’ |
| ΔbHLH-C-Fwd | 5’CGGAGGCGGCACCCAGTCGCTGAACG 3’ |
| ΔC-Rev | 5’AGCAGTGATCCGCGTCCCTGGCAGCTTGCCATCTTGGAGTC 3’ |
| T2A-Top | 5’AACCGTCAGATCCGCTAGCAGGGACGCGGATCACTGCTTACTTGCGGGGATGTG GAGGAGAATCCCGGTCCCCCGCTACCGGTCGCCACC 3’ |
| T2A-Bottom | 5’GGTGGCGACCGGTAGCGGGGGACCGGGATTCTCCTCCACATCCCCGCAAGTAA GCAGTGATCCGCGTCCCTGCTAGCGGATCTGACGGTT 3’ |
| Twist1-pWPI-Fwd | 5’TATCGATCACGAGACTAGCCTCGAGGTTTGGAACAAAAGTTGATTTCTGAAGAA GATTT 3’ |
| Twist1-pWPI-Rev | 5’CCGCCGCTTCCGCCGGAGCTGCCGCCTCCGGATCCGTGGGACGCGGACATGG 3’ |
| TT-Fwd | 5’GGCGGATCTGGCGGAGGATCTGGGAGTTTATGATGCAGGACGTGTCCAG 3’ |
| TT-Rev | 5’GGGGGGCGGAATTCCTGCAGCCCGTAGTTTTCAGTGGGACGCGGACA 3’ |
| E47-Fwd | 5’GGCGGATCTGGCGGAGGATCTGGAGAGTTTATGAACCAGCCGCAGAGGA 3’ |
| E47-Rev | 5’GGGGGGCGGAATTCCTGCAGCCCGTAGTTTTCACATGTGCCCGGCG 3’ |
| SDM-Fwd | 5’TCCTACTCCGgCAGCTCCCGGC 3’ |
| SDM-Rev | 5’GGGGTAGTACTGGGAGGTCC 3’ |
| E12-Fwd | 5’ TGGCGGAGGATCTGGAGAGTTTATGGCGCCTGTGGG 3’ |
| E12-Rev | 5’GGGGGGCGGAATTCCTGCAGCCCGTAGTTTTCACATGTGCCCGGCG 3’ |
