# Supplementary figures and images for: Functional analysis of TWIST1 domains regulating smooth muscle cell phenotype
Source: Front Cardiovasc Med. 2025 Oct 31;12:1659847. doi: 10.3389/fcvm.2025.1659847 (PMC12615416; doi:10.3389/fcvm.2025.1659847)

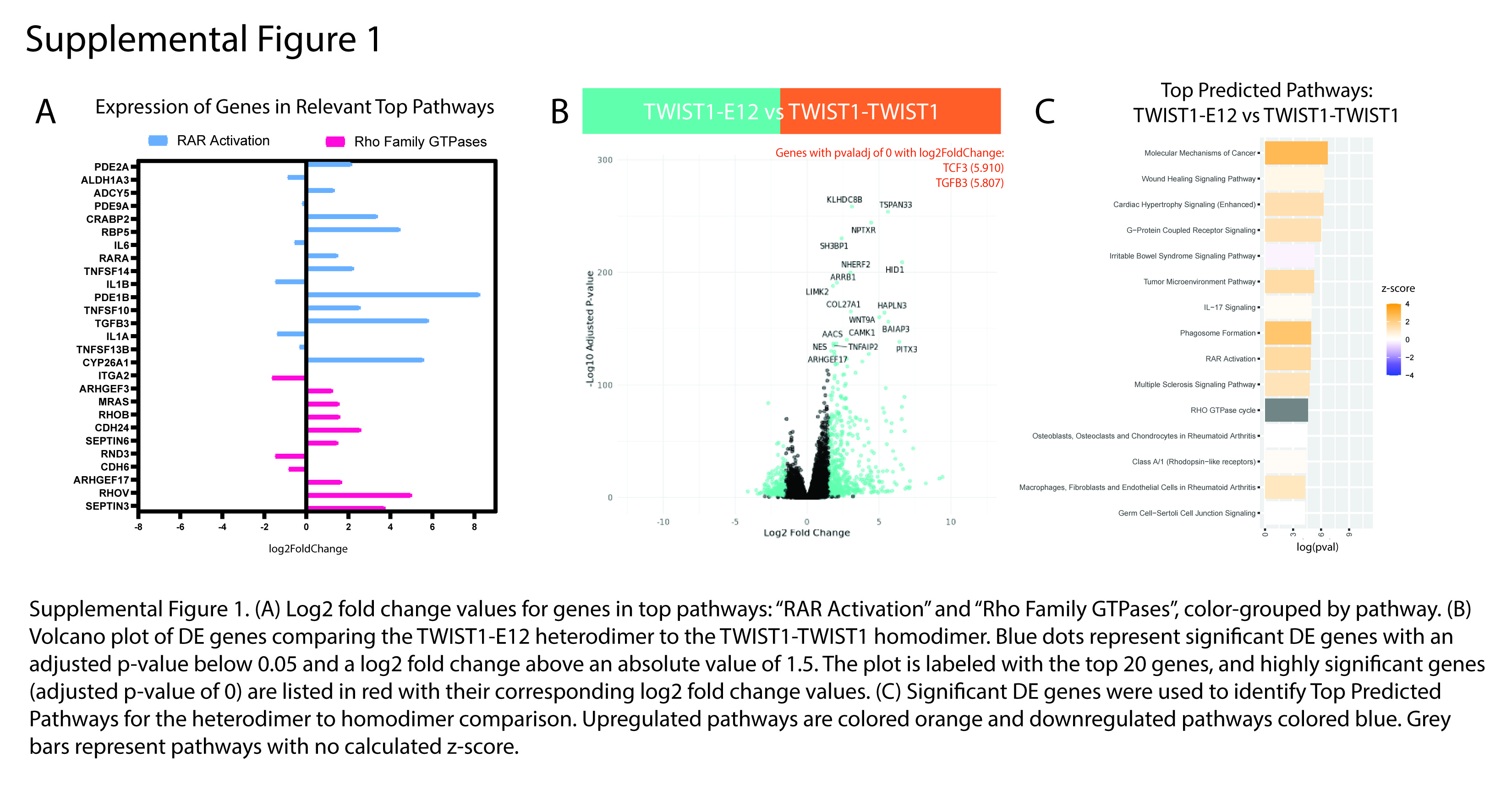

Supplement: Supplementary file 5 [file Image1.jpeg]

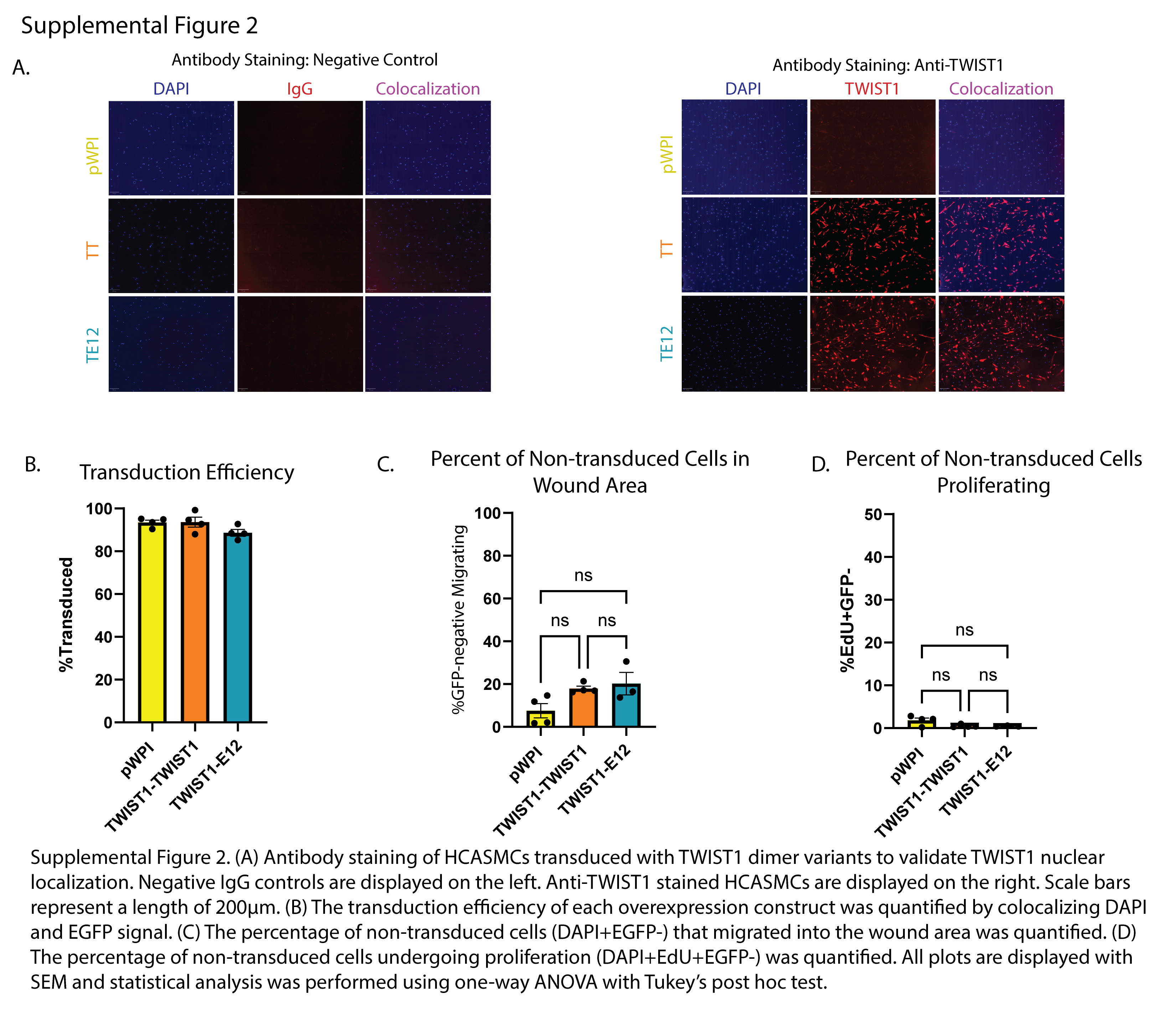

Supplement: Supplementary file 6 [file Image2.png]

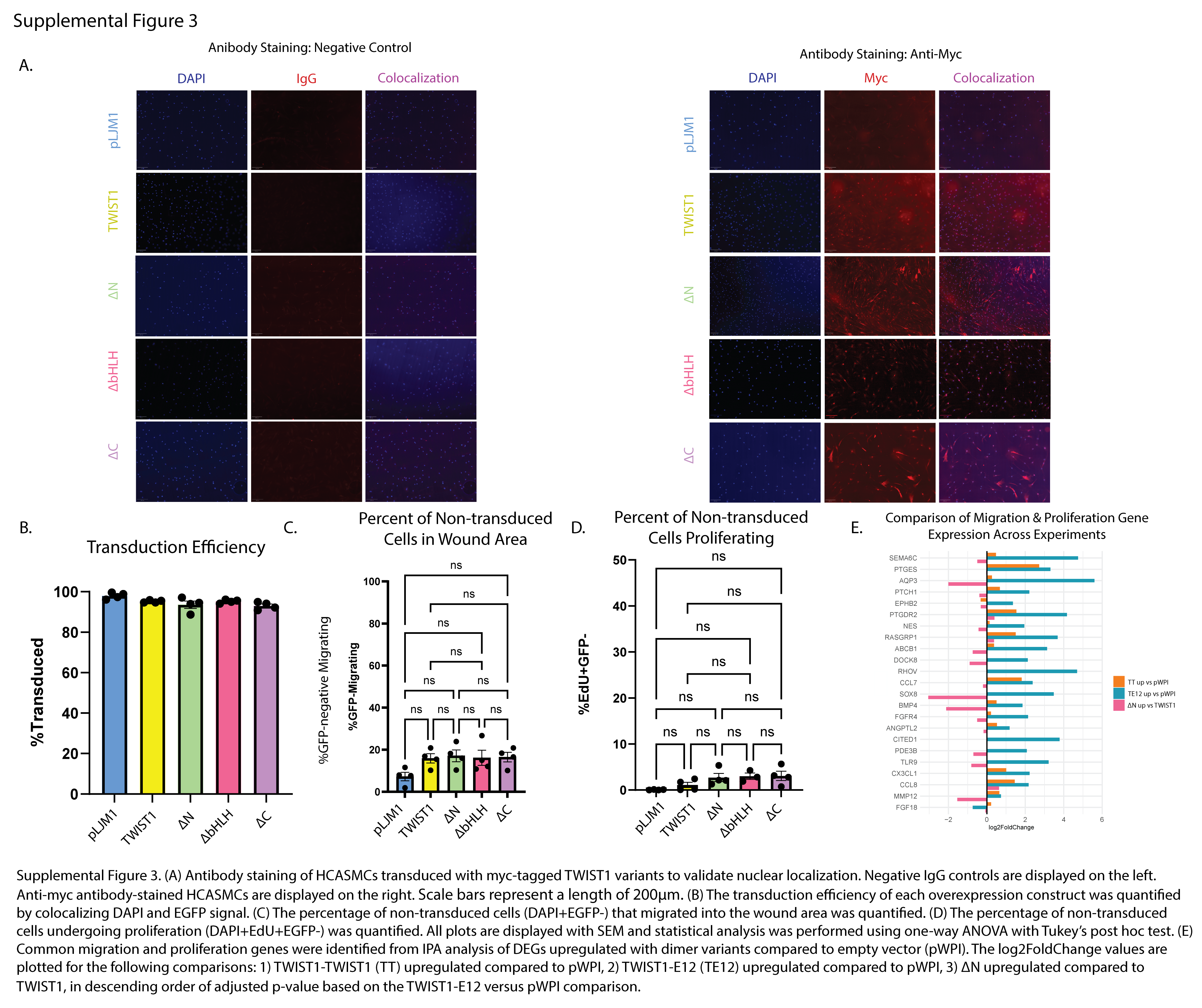

Supplement: Supplementary file 7 [file Image3.png]

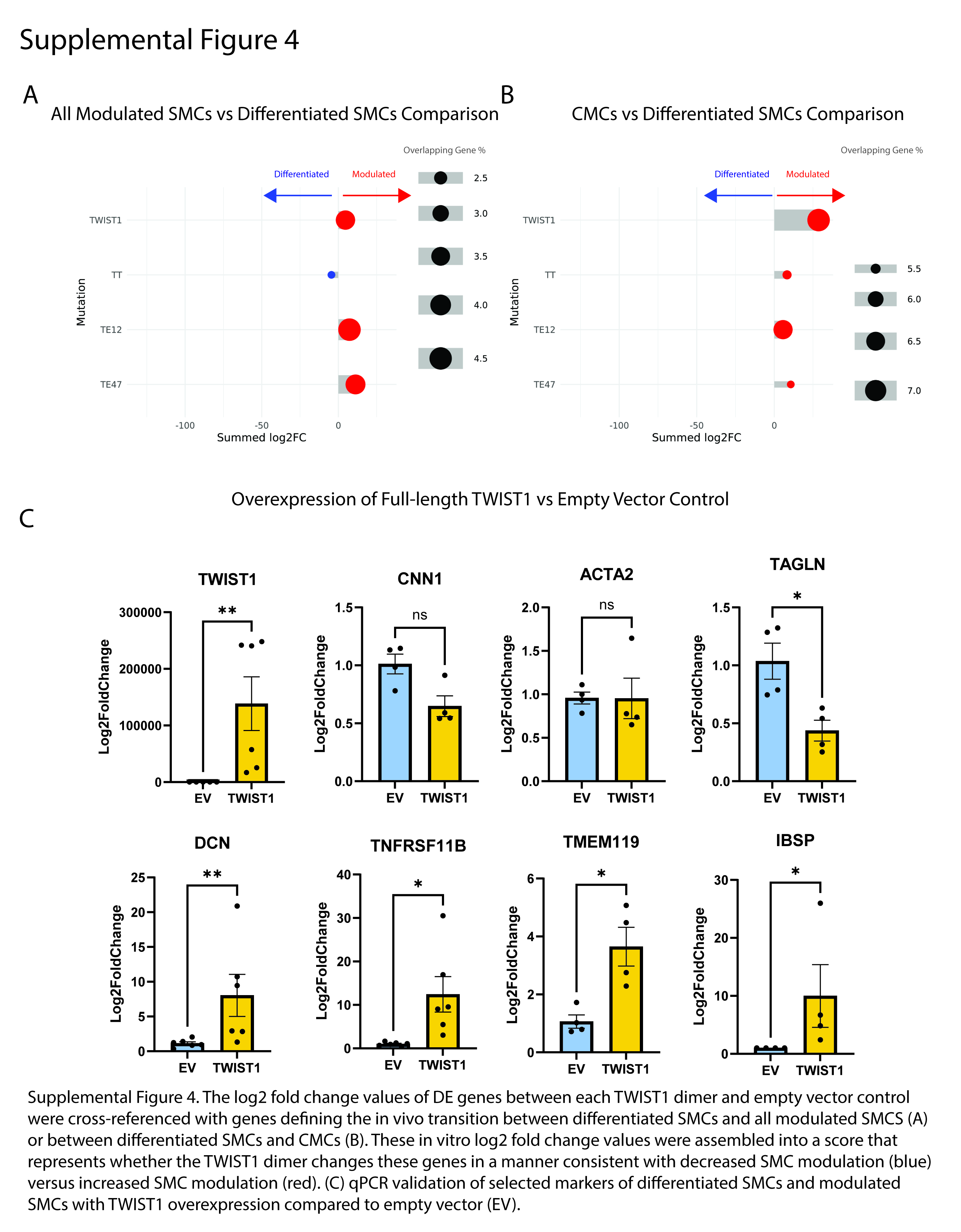

Supplement: Supplementary file 8 [file Image4.jpeg]
